# Returning to a Normal Life via COVID-19 Vaccines in the United States: A Large-scale Agent-Based Simulation Study

**DOI:** 10.2196/27419

**Published:** 2021-04-29

**Authors:** Junjiang Li, Philippe Giabbanelli

**Affiliations:** 1 Department of Computer Science & Software Engineering Miami University Oxford, OH United States

**Keywords:** agent-based model, cloud-based simulations, COVID-19, large-scale simulations, vaccine, model, simulation, United States, agent-based, effective, willingness, capacity, plan, strategy, outcome, interaction, intervention, scenario, impact

## Abstract

**Background:**

In 2020, COVID-19 has claimed more than 300,000 deaths in the United States alone. Although nonpharmaceutical interventions were implemented by federal and state governments in the United States, these efforts have failed to contain the virus. Following the Food and Drug Administration's approval of two COVID-19 vaccines, however, the hope for the return to normalcy has been renewed. This hope rests on an unprecedented nationwide vaccine campaign, which faces many logistical challenges and is also contingent on several factors whose values are currently unknown.

**Objective:**

We study the effectiveness of a nationwide vaccine campaign in response to different vaccine efficacies, the willingness of the population to be vaccinated, and the daily vaccine capacity under two different federal plans. To characterize the possible outcomes most accurately, we also account for the interactions between nonpharmaceutical interventions and vaccines through 6 scenarios that capture a range of possible impacts from nonpharmaceutical interventions.

**Methods:**

We used large-scale, cloud-based, agent-based simulations by implementing the vaccination campaign using COVASIM, an open-source agent-based model for COVID-19 that has been used in several peer-reviewed studies and accounts for individual heterogeneity and a multiplicity of contact networks. Several modifications to the parameters and simulation logic were made to better align the model with current evidence. We chose 6 nonpharmaceutical intervention scenarios and applied the vaccination intervention following both the plan proposed by Operation Warp Speed (former Trump administration) and the plan of one million vaccines per day, proposed by the Biden administration. We accounted for unknowns in vaccine efficacies and levels of population compliance by varying both parameters. For each experiment, the cumulative infection growth was fitted to a logistic growth model, and the carrying capacities and the growth rates were recorded.

**Results:**

For both vaccination plans and all nonpharmaceutical intervention scenarios, the presence of the vaccine intervention considerably lowers the total number of infections when life returns to normal, even when the population compliance to vaccines is as low as 20%. We noted an unintended consequence; given the vaccine availability estimates under both federal plans and the focus on vaccinating individuals by age categories, a significant reduction in nonpharmaceutical interventions results in a counterintuitive situation in which higher vaccine compliance then leads to more total infections.

**Conclusions:**

Although potent, vaccines alone cannot effectively end the pandemic given the current availability estimates and the adopted vaccination strategy. Nonpharmaceutical interventions need to continue and be enforced to ensure high compliance so that the rate of immunity established by vaccination outpaces that induced by infections.

## Introduction

The Centers for Disease Control and Prevention (CDC) forecasted that 300,000 deaths would be attributable to COVID-19 by the end of the year. Reality defied expectations, as COVID-19 was *directly* responsible for approximately 350,000 deaths in the United States out of 20 million *reported* cases (for forecasts and total case numbers, see [1]), which may only represent one out of seven actual cases based on CDC estimates for September 2020 [2]. Despite popular comparison with the flu, the ongoing COVID-19 epidemic has thus already claimed five times as many lives than the worst year for the flu, whose recent yearly death tolls range from a low of 16,000 to a high of 68,000 [3]. To contextualize the impact of COVID-19, we noted that the US *life expectancy* decreased by more than a year, which is ten times worse than the decline from the opioid epidemic [4]. In another comparison, 2020 is the *largest single-year increase in mortality* in the United States since 1918, which had both a flu pandemic and a war. This reflects both direct and *indirect* consequences of COVID-19, such as disrupting in-person treatments [5] and supply networks, with effects as far ranging as an increase in drug overdose [6]. To complement measures of short-term effects such as deaths or number of cases, we also noted the long-term impacts captured by the outpatient journey. Common symptoms often persist over a month (eg, fatigue, cough, headache, sore throat, or loss of smell) [7-9], and less frequent ones can be severe since COVID-19 involves many organs. Effects can involve the cardiovascular system in up to 20%-30% of patients who are hospitalized [10,11] (eg, cardiac injury, vascular dysfunction, or thrombosis), result in kidney injury [10] or pulmonary abnormalities [12], or lead to a deterioration in cognition due to cerebral microstructural changes [13]. Based on similar infections, such effects can be long: for instance, inflammation of the heart caused by viral infections (eg, myocarditis) can have a recovery period spanning months to years.

Interventions in 2020 were strictly *nonpharmaceutical*, as vaccines were being developed and tested. Such intervention strategies have included preventative care (eg, social distancing, handwashing, and face masks), lockdowns (eg, travel restrictions, school closures, and remote work), and logistics associated with testing (eg, contact tracing and quarantine) [14,15]. The range of nonpharmaceutical interventions adopted at various times across countries can be seen in further details through the CoronaNet project [16] or the collection of essays “mobilizing policy (in)capacity to fight COVID-19” published in mid-2020 [17]. In early 2021, two vaccines were deployed (Pfizer-BioNTech and Moderna) with plans for up to three additional vaccines (AstraZeneca, Janssen, and Novavax) [18]. With the availability of vaccines comes the key question: when will life return to normal in the United States? The implicit expectation is to see a return to normalcy thanks to the vaccine, rather than due to a high number of cases with its accompanying death toll.

In a highly publicized interview, Dr Anthony Fauci, director of the National Institute of Allergy and Infectious Diseases, estimated a return to normal by fall, *if the vaccination campaign is successful* [19]. Getting a precise estimate of when life will return to normal is a challenge, as it depends on numerous interrelated factors: potential behavioral changes affecting nonpharmaceutical approaches (eg, lesser compliance to mask wearing and social distancing), participation in the vaccination campaign, logistics associated with vaccination (ie, who can get vaccinated and when), and mutations leading to new strains with different biological properties (eg, higher infectivity) or unknown vaccine responses. In this paper, we use large-scale simulations to identify *when* there will be an inflection point in the dynamics of the disease and the *level* of cases that will be obtained.

Simulations have been used since the early days of the COVID-19 pandemic. Classic compartmental epidemiological models were first produced (eg, many susceptible-exposed-infected-removed models [20-23]), with a focus on estimating broad trends and key epidemiological quantities such as the expected number of new cases generated by each infected individual (ie, the basic reproduction number R_0_). Such compartmental models provide limited support to study the effect of interventions, for instance by lowering the contact rate to represent the impact of social distancing. A research shift in the second part of 2020 resulted in the growing use of *agent-based models (ABMs)* to support the analysis of interventions by explicitly modeling each individual and their interactions among each other or with the environment. This shift to individual-level models was underpinned by the evidence of *heterogeneity* in risk factors (eg, older age, hypertension, respiratory disease, and cardiovascular disease [24,25]) and behaviors (eg, noncompliance with social distancing orders) based on personal beliefs and values [26,27]. There is also spatial variation in socio-ecological vulnerability to COVID-19 [28], with rural counties being at higher risk (due to eg, older population with more underlying conditions and lower access to resources) [29,30] and hence experiencing higher mortality rates [31]. Finally, there is a documented heterogeneity in transmission based on contact tracing data [32], which stresses the need to use realistic networks when modeling the spread of COVID-19 [33]. Considering this growing evidence base, our study relies on an ABM, which accounts for individual heterogeneity (eg, in age), explicitly embeds them in a network to model their contacts, and simultaneously considers different network types (eg, community and work) to account for various settings.

By adding vaccines to a previously validated ABM of COVID-19, we are able to assess how the number and timing of cases depends on key factors such as the population’s interest in vaccines and the efficacy of vaccines. Our specific contributions are twofold:

1. We extend the validated COVASIM model with a detailed process of vaccination, accounting for vaccine efficacy, interest in vaccination, and fluctuations in vaccination capacity. Our process models the need for two doses and the possibility of being infected until the second dose is administered.
2. We examine vaccination interventions under two hypotheses for the number of doses available and considering concurrent nonpharmaceutical interventions.

The remainder of this paper is structured as follows. In our methods, we briefly cover the rationale for choosing COVASIM and how we adapted the model to account for the latest epidemiological evidence. We then explain which nonpharmaceutical interventions are simulated, in line with our previous work [34]. Most importantly, we detail the novel extension of vaccines into COVASIM and our examination of the trends in cumulative infections using a logistic growth model. The following section presents and analyzes our results. Our final section discusses our main findings and provides an exhaustive list of limitations due to the ongoing nature of the pandemic and challenges in vaccination.

## Methods

### Overview

**Figure 1:**
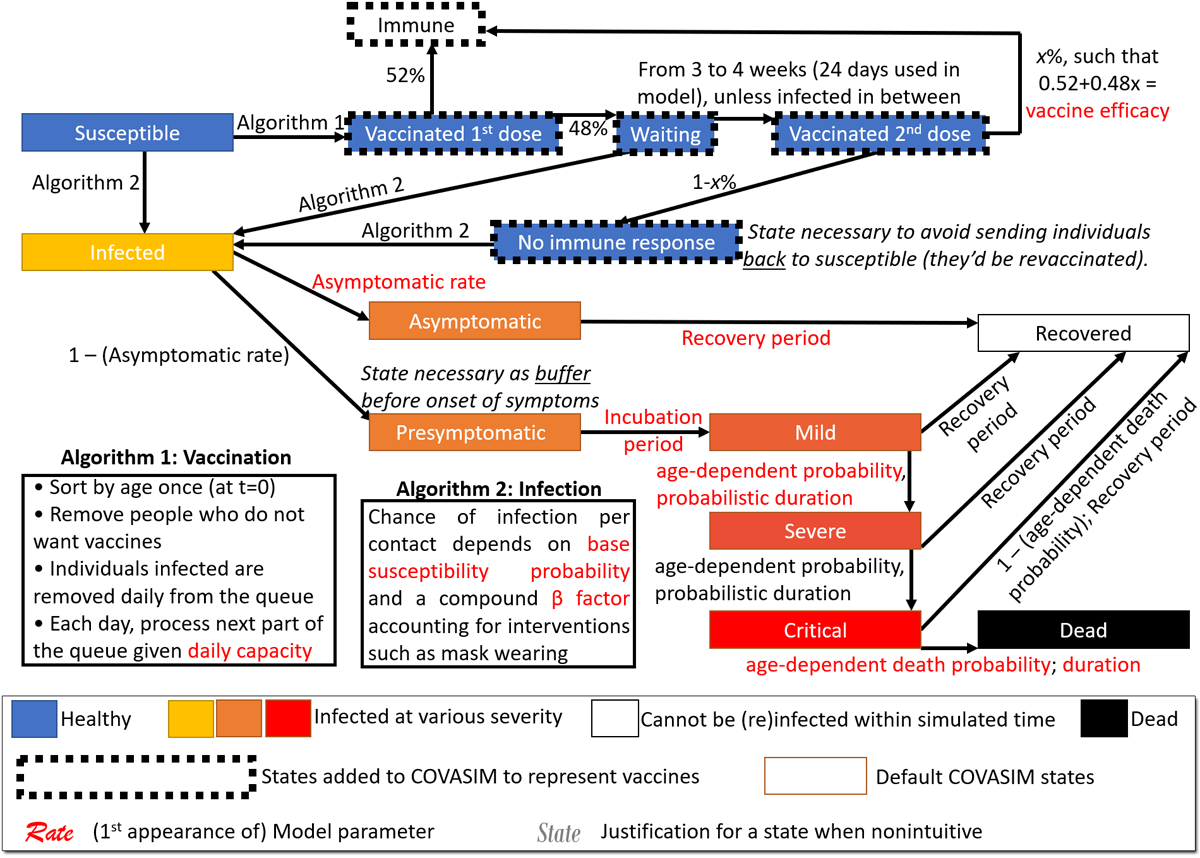
Overview of our modified COVASIM model containing the state diagram and specification of all transitions, including key procedures for vaccination and infection.

COVASIM was developed under leadership of the Institute for Disease Modeling and released in May 2020 by Kerr and colleagues [35]. It is one of several open-source ABMs, together with OpenABM-Covid19 [36] or COMOKIT [37]. The model captures the transition from susceptible to infected followed by a split between asymptomatic individuals and various degrees of symptoms, resulting either in recovery or death (Figure 1). The model was created to support interventions offered at the time, which did not include vaccination. We thus *modified the model* to account for our current understanding of viral dynamics and the use of vaccines over two doses (Figure 1). When instantiating the model to the US population, we used a resolution of 1:500 (ie, each simulated agent accounts for 500 US inhabitants). Given our resolution and target population size, our application exceeded half a million agents and can thus be described as a “*large-scale COVID-19 simulation*” [38]. Our simulations started on January 1, 2020, using CDC data for the number of infected, recovered, and immunized individuals to date (see subsection Initializing the Model). We then simulate for 6 months, that is, 180 time ticks based on a temporal resolution of 1 day per simulation step (ie, *tick*). To cope with the computational challenges created by a large-scale stochastic model, a philanthropic grant supports us in performing cloud-based simulations via the Microsoft Azure (Microsoft Corporation) platform.

### The COVASIM Model: Rationale for Selection and Evidence-Based Updates

Apart from being open source, there are two reasons that we selected COVASIM. First, it captures heterogeneity within individuals (eg, assigns an age and uses age-specific disease outcomes) and transmission patterns by placing agents within synthetic networks corresponding to a multiplicity of contexts: work (based on employment rates), school (based on enrollment), home (based on household size), and the general community. However, these high-resolution age-specific contact patterns are not unique to COVASIM. For example, the OpenABM-Covid19 [36] also embeds agents in age-stratified occupation networks (encompassing work and school), household networks, and a *general* random network. COMOKIT [37] similarly uses the Gen* toolkit from the same team to redistribute populations from census units down to exact buildings such as the nearest school. Thus, the second rationale for choosing this platform is that it has been used in the most peer-reviewed modeling studies to date [39,40], hence providing an additional layer of scrutiny and confidence in the correctness of the model (ie, validation) and its implementation (ie, verification). As detailed in our recent study [34], changes in the evidence base have required alteration in the model to keep it valid. Consequently, we modified three COVASIM parameters to account for the current biological and epidemiological evidence on COVID-19 (Table 1).

**Table 1:** Adjusted parameters based on reports in the United States.

COVASIM construct	Initial value	Modified value	Rationale for modification
Incubation: delay from infection to viral shedding	Lognormal(4.6, 4.8)	Lognormal(4.1, 4.8)	The combined distribution of the incubation period did not match the latest evidence. The adjustment aligns it with the evidence.
Incubation: delay from viral shedding to onset of symptoms	Lognormal(1,1)	Lognormal(1, 1.8)	Same as above
Proportion of symptomatic cases	0.7	0.6	Although reports vary, Dr Fauci stated that 40% of the US cases were asymptomatic.

### Selection and Representation of Concurrent Nonpharmaceutical Interventions

In addition to support for heterogeneity, COVASIM implements several nonpharmaceutical interventions. Although our focus is on vaccines, such interventions may be continuing in parallel with the vaccination campaign; hence, we have to take them into account when forecasting case counts. Interventions can be organized into three broad categories: preventative care (eg, *social distancing* and *face masks*), lockdown (eg, *stay-at-home* orders such as remote work or school closures), or testing-related (eg, *testing* itself, then *quarantining* and *contact tracing*) [14,41,42]. In line with our previous work on nonpharmaceutical interventions, we considered all 6 specific interventions. Although all 6 are natively supported by the COVASIM platform, we changed testing delays from their default value (constant) to a distribution (based on a survey across all 50 US states) [43], thus accounting for the variability observed in practice.

**Figure 2:**
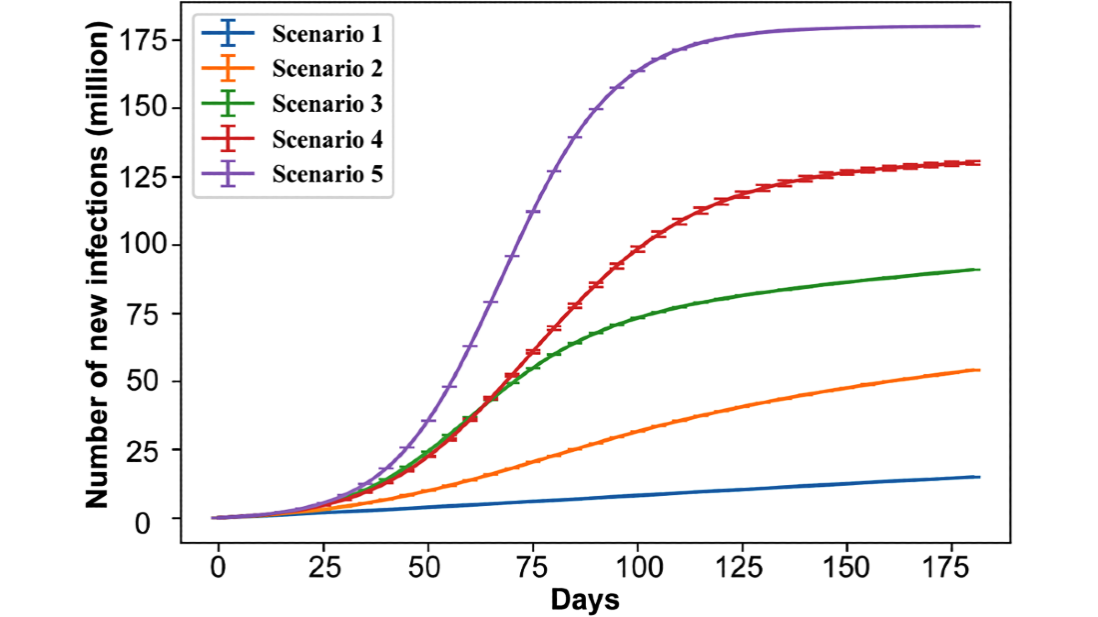
Number of new infections during the simulation (ie, cumulative cases) under five scenarios (each based on a combination of interventions), which were selected for their ability to represent different trends in the number of cases over time, without a vaccine.

Since our focus is on vaccines, our search space is primarily devoted to quantifying the effect of vaccine-related variables (ie, efficacy, compliance, and capacity). As every nonpharmaceutical intervention could lead to several variables (eg, compliance with face masks or efficacy of face masks), considering all variables for every such intervention *in addition to* vaccine-related variables would lead to an impractical search space. We thus leveraged the systematic assessment of our previous study [34], which simulated all combinations of nonpharmaceutical interventions at two different levels of strength (ie, a binary factorial design of experiments). We analyzed results from this broad search to select 5 scenarios (Table 2) that resulted in five different levels of infection count after 6 months, in the absence of any vaccine (Figure 2). In other words, to circumvent the unwieldy notion of simulating all aspects of vaccines and nonpharmaceutical interventions, we selected 5 scenarios that produce linear to logistic growths in cumulative infections, thereby conducting a parameter sweep across possible growth behaviors. We supplemented these 5 scenarios with an extreme *no intervention* scenario, which provides an upper bound on the number of cases.

**Table 2:** Scenarios depicting concurrent nonpharmaceutical interventions, chosen for their ability to create five markedly different outcomes together with a nonintervention case.

Features	Scenario					
	1	2	3	4	5	6 (do nothing)
Networks impacted	Work, school	Work, school	Community	Community	Community	All
Contact in work and school (as a function of default; %)	70	95	N/A^a^	N/A	N/A	100
Contact in community (as a function of default; %)	N/A	N/A	70	70	90	100
Daily tests^b^	1,110,000	600,000	600,000	1,110,000	600,000	No testing
A positive test leads to quarantine. Is a second test required to end quarantine?	No	Yes	No	Yes	Yes	No testing
Test sensitivity	1	1	1	0.55	0.55	No testing
Ratio of contacts that can be traced	0.2	1	1	0.2	0.2	No tracing
After how many days will contact tracing results arrive (ie, contact tracing delay)?	0	7	7	7	7	No tracing
Starting contact tracing if one has just been tested and exposed (one infected peer)	Yes	No	No	Yes	No	No tracing

^a^N/A: not applicable.

^b^These numbers reflect the total daily capacity at the scale of the US population. As our simulation uses a scale of 1:500, the capacity in the model is scaled down accordingly.

**Figure 3:**
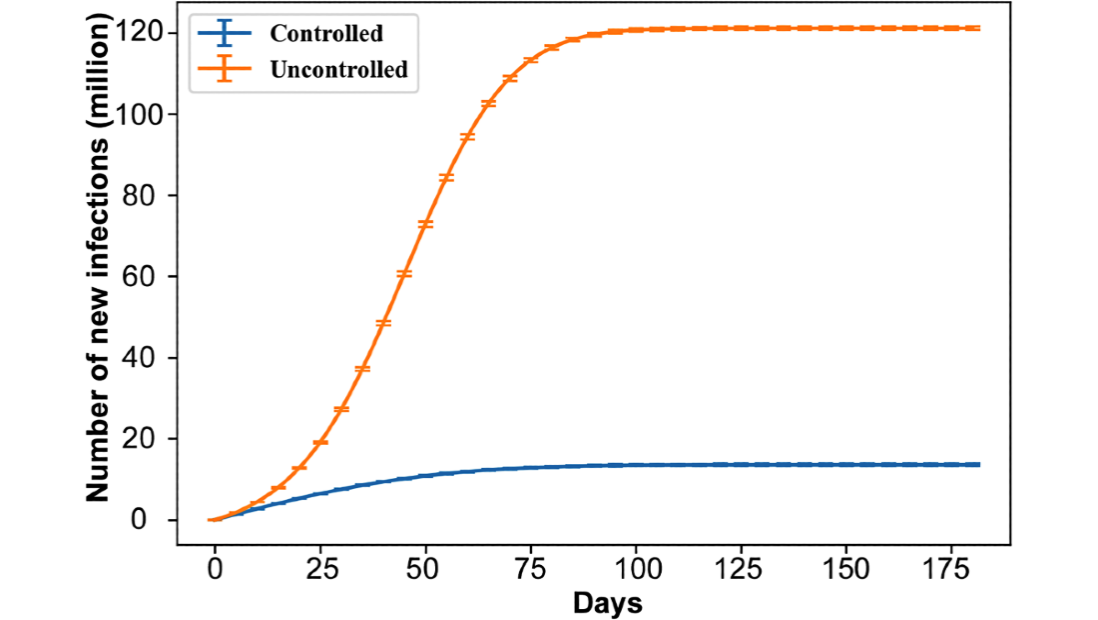
Comparison of changes in cumulative infections between a COVASIM simulation and reality from September 3, 2020, to the end of 2020. The simulation included a reduction on work and school contacts (set to 95% of their capacity), 600,000 daily and highly sensitive tests, quarantining upon testing, immediate tracing to identify all contacts, and a presumptive approach.

Given that we made minor changes to the biology (incubation and proportion of symptomatic cases) and consider several ongoing intervention scenarios, it is necessary to confirm the validity of the model established using earlier data in previously published studies. Consequently, we ran the modified COVASIM model based on data observed until September 3, 2020, and compared the simulated results with observations until the end of year. Similar trends and orders of magnitude were observed (Figure 3), thus providing qualitative validation. Note that the 5 scenarios chosen (Table 2) bound the growth of COVID-19 in the United States such that we are comprehensively examining possible trends going forward instead of limiting ourselves to the single trend that fit best on previous data.

### Extending COVASIM With Pharmaceutical Interventions: A Two-step Vaccination

As detailed in our discussion, there is substantial uncertainty and frequent changes regarding the number of vaccines that *may* be administered monthly. We thus considered two vaccine availability scenarios, both proposed by federal governments. The first scenario from the former Trump administration, named Operation Warp Speed, stated that vaccines will be available in tiered amounts (20 million in December, 30 million in January, and 50 million every month thereafter). The second scenario from the Biden administration, known as the *100-day goal*, proposes that there will be 1 million vaccines every day [44], thus covering 50 million Americans. Although there are other scenarios, they vary from state to state (eg, the governor of New Jersey aspires to vaccinate 70% of the adult population within 6 months [45]) and are subject to frequent revisions. Given the countrywide nature of our simulation, we relied on federal plans while detailing challenges (see also the Discussion section).

In setting the monthly capacity, we noticed the necessity to *adjust the schedule* of the Operation Warp Speed plan, since the initial aim of 20 million people immunized by the end of December 2020 only resulted in 3 million doses administered. In other words, it would be incorrect to model the monthly capacity of Operation Warp Speed as announced since there is evidence that its initial objective was unmet, due to a variety of logistical challenges. Consequently, we shifted the expectations of the Operation Warp Speed plan by 1 month, such that the capacity for January now corresponds to the initial expectations for December (20 million) and so forth.

At the same time as either vaccination schedule is active, we also have the 6 scenarios listed in the previous sections. As these scenarios include a no-intervention case, we are able to study the interaction between nonpharmaceutical interventions and vaccines. In total, this gives 12 distinct situations. In addition, we also varied two essential parameters regarding vaccines: the percentage of the population that seeks vaccination (which we refer to as *vaccine compliance* from hereon) and the efficacy of the vaccine. Varying these two parameters across 12 situations in a large-scale ABM results in substantial computing needs. These are challenging to parallelize, as the run time of each experiment is not the same. Therefore, we took advantage of the massive parallelism enabled by the cloud computing platform Azure to accelerate computation. Using this platform, we varied vaccine compliance and vaccine efficacy between the bounds listed in Table 3.

**Table 3:** Vaccine parameters used in the study. Intermediate values in the interval bounded by the low and high values are automatically explored.

Parameters	Low value (%)	High value (%)
Vaccine compliance	20	60
Vaccine efficacy	88	99

Regarding our approach to vaccine efficacy, we noted that individuals can be infected after their first dose, as has been documented on thousands of cases [46]. We thus used the probability of 52% (observed in clinical trials [47]) to obtain early protection by the vaccine, and otherwise, an individual may still be infected in the waiting period leading to the second dose. After the second dose is applied, we needed to ensure that the agent meets the vaccine efficacy set by our parameters. That is, the probability of obtaining immunity after the second dose was set such that the probability of immunity *from the two doses* matches the vaccine efficacy.

Although we did not track which of the two approved mRNA COVID-19 vaccines (Pfizer-BioNTech or Moderna) were administered, we varied vaccine efficacy to account for a margin of uncertainty regarding their respective performances. Since the vaccine capacity is either planned to increase (Operation Warp Speed) or be at a high constant rate, a simulated agent given one dose will always be able to come back to get the second dose on time. Should an agent be contaminated or die before the second dose, it is then released for administration to another agent.

We also varied the percentage of the population who seeks vaccination. As noted in a recent study, this percentage has varied among studies: 10.8% did not intend to be vaccinated when asked in April 2020, but this number jumped to 31.1% by May, and an August poll found that only a *minority* would want to be vaccinated [48]. In addition to changes in the sociopolitical climate and public discourse surrounding vaccination, there will also be changes since “many receptive participants preferred to wait until others have taken the vaccine” [49]. Seeing positive vaccination outcomes in others may in part address the fear of serious side effects, which is a recurring concern for individuals who may not intend to participate in vaccination [50]. Given past variations and changes in the future, we handled uncertainty through a parameter sweep in vaccine compliance.

### Initializing the Model

A simulation model is composed of an initialization (setting characteristics of agents for t=0) and rules governing its update, thereby producing data for analysis. The previous subsections covered the rationale for the inclusion of agents’ characteristics and the design of the rules, while the next subsection focuses on the analysis. This subsection thus briefly covers our approach to initialization such that our results could be independently replicated by other modeling teams.

Our initial time tick t=0 corresponds to January 1, 2020. We thus needed to set the number of agents who have been infected, recovered, or immunized (due to the rollout of vaccines in December) by that time. A COVID-19 case remains infectious within a time window of 2 weeks, after which there is either recovery or complications. From December 18-31, there was a total of 3,311,345 active cases. To appropriately initialize our simulation, we needed to further track *when* an individual was infected. Incorrectly setting them to be all infected on December 18 would result in nobody being infected when the simulation starts on January 1. At the other extreme, assuming that they were all infected on December 31 would lead to an overestimate of disease spread into 2021. We thus seeded the timing of each infection by using the daily distribution from CDC data between December 18-31 (Table 4). All numbers were divided by 500 since our agent resolution is 1 agent for 500 real-world US inhabitants (1:500). The number of individuals who acquired immunity via recovery was set to the total case count observed by December 17. Individuals who died from COVID-19 are grouped together with recovered ones (ie, we did not subtract them from the count) since our simulations track the number of new infections; dead individuals do not alter these results as they can neither be infected nor infect others. The total number of individuals immunized from vaccination was set to 2 million (ie, 4000 agents).

**Table 4:** Timing of the infection in the 2 weeks preceding the start of our simulation, such that our agents can be initialized at the appropriate state of their infection.

Specific day of the infection	Individuals infected, n
December 18, 2020	236,063
December 19, 2020	202,050
December 20, 2020	198,129
December 21, 2020	184,632
December 22, 2020	196,516
December 23, 2020	229,746
December 24, 2020	193,277
December 25, 2020	139,152
December 26, 2020	179,707
December 27, 2020	146,593
December 28, 2020	177,814
December 29, 2020	201,428
December 30, 2020	230,982
December 31, 2020	229,634

### Analyzing the Progression of Cumulative Infections Through a Logistic Growth Model

To quantify the spread of the disease, we fitted the progression of cumulative infection to a logistic growth model, which is a simple yet effective model describing resource-limited growths in natural processes and has been used on several occasions for COVID-19 [51-53]. Let the cumulative infection be *P* = *P*(*t*), then the logistic model stipulates that *P* is the solution of the differential equation:



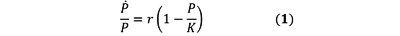



where 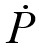 is the time derivative of *P*, *r* is the *growth rate* (proportional to the maximum value attained by 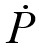), and *K* is the *carrying capacity*. As our simulations produce the complete time series for *P*, we can estimate 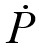 using finite differences, thereby extracting parameters *r* and *K* through a linear regression as equation 1 suggests. In the regression, the independent and dependent variables are *P* and 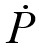 / *P*, respectively. In addition, we measured the goodness of fit as that of the linear regression. Since the simulation is stochastic, multiple replications are needed for each configuration to obtain an average behavior. We used the CI method [54] to perform enough replications so that for every time step *t*, the 95% CI of *P* at time *t* falls within 5% of the average. Therefore, we performed the fitting for each individual run and computed the average *r* and *K* across all runs.

Although we report the carrying capacity *K* in Multimedia Appendices 1 and 2, the interpretation of this variable can be difficult for a broader audience. The growth rate *r* is *proportional* to the maximum *fraction of the carrying capacity K* that is infected on the worst day. In other words, it is an indication of how fast the disease spreads at its peak, based on another variable. For ease of interpretation, we focused on the adjusted growth rate whose unit is directly in number of individuals. The adjusted growth rate reported in this paper is obtained as:



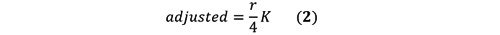



For instance, an adjusted value of 200,000 means that at most 200,000 individuals will be infected on the worst day.

**Figure 4:**
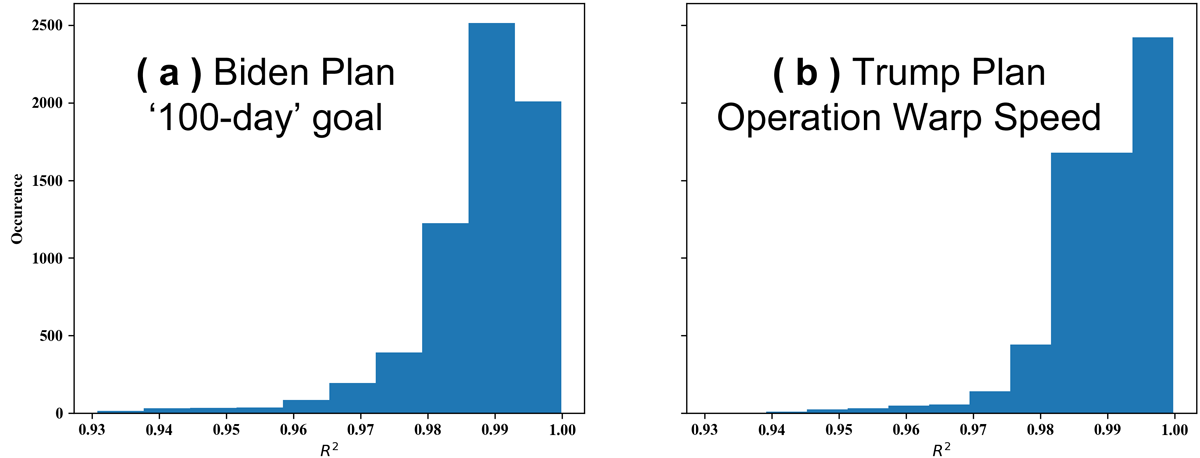
Distributions of the average goodness of fit R^2^ for each vaccination plan, demonstrating the validity of fitting logistic growth models from t≥20.

As the early steps of the simulation witness a shift from a vaccine-naïve population to one that gradually builds vaccine-based immunity, early trends differ from the longer ones that are the focus of this study. This is a typical situation in modeling, whereby estimating the long run performance measures requires to first run the model for a certain amount of time (known as the *warm-up period*) [55]. We empirically determined that a warm-up period of 20 days was sufficient to start the curve fitting; that is, we created the time series for *P* starting from *t*≥20. As evidenced by Figure 4, this warm-up period results in very good fit for the logistic model under both federal plans. This approach also generalizes better, since the reported *r* and *K* can accurately characterize the spread of the disease for most time periods instead of being skewed by the first few days.

**Figure 5:**
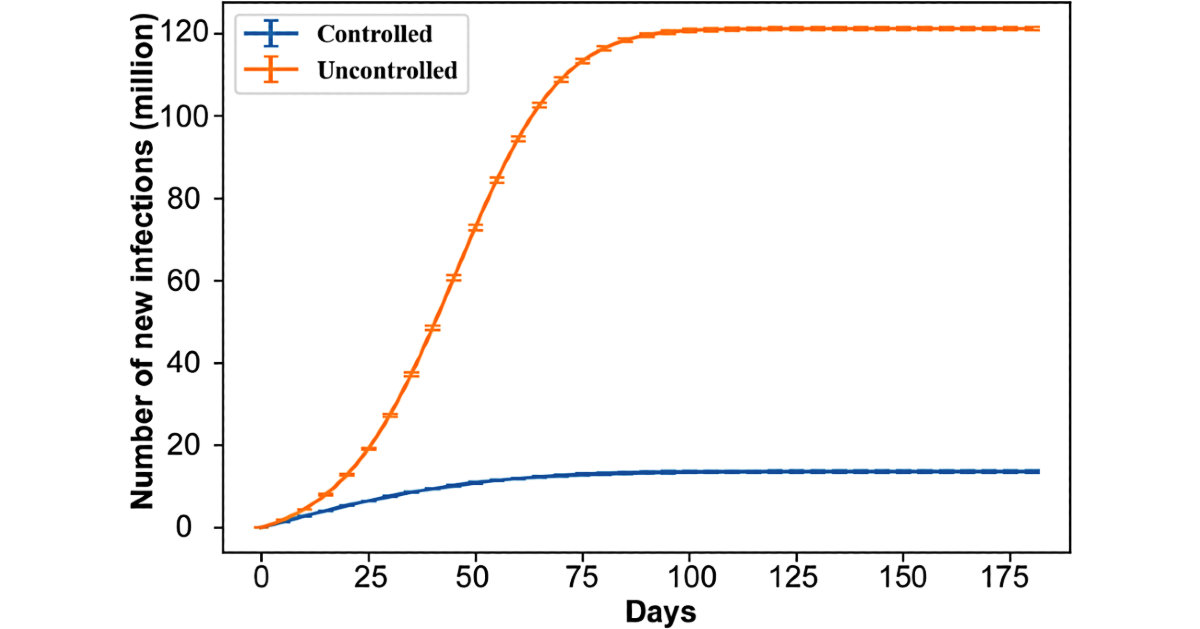
Number of new infections during the simulation (ie, cumulative cases) under Operation Warp Speed with vaccine compliance of 0.6, vaccine efficacy of 0.99, scenario 1 for nonpharmaceutical interventions (“controlled” case: blue), and scenario 6 consisting of no interventions (“uncontrolled” case: orange).

An essential aspect of a return to normalcy is about the *conditions* under which that is achieved. If the disease is left uncontrolled, and simplifying the matter of variants, we would still return to *normalcy* within 6 months because a large share of the population would already have been infected and either recovered or died (Figure 5). The goal is thus not *only* to eventually achieve stability in the number of cases but to achieve it at a minimal level (Figure 5; bottom blue curve).

### Simulation Management in Azure

**Figure 6:**
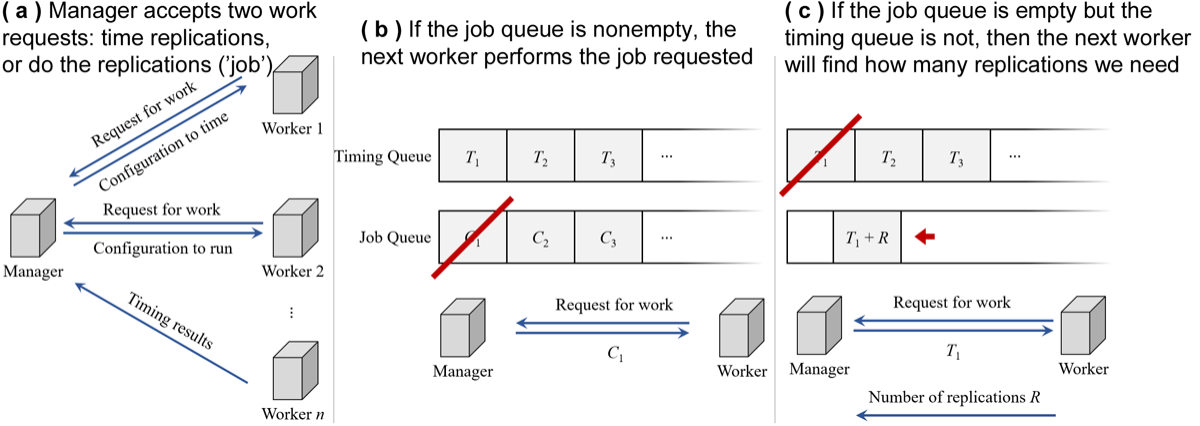
Our simulation management architecture to leverage parallelism on Microsoft Azure.

To efficiently orchestrate simulations over the Microsoft Azure cloud computing platform, we used a distributed scheme shown in Figure 6. The setup starts by creating a manager, which uses queues to organize the two types of work that need to be performed.

1. Given a configuration (eg, which scenario, compliance level, and vaccine efficacy), they need to determine how many replications are necessary for a tight CI of 95%. These tasks are tracked in the timing queue.
2. Given a configuration and set number of replications, perform the computations to produce the results. These tasks are tracked in the job queue.

Available workers contact the manager, who will assign work (Figure 6a) by prioritizing the job queue and then the timing queue. For example, if a worker notifies the manager that it is available and there is a simulation run to perform in the job queue, then the manager will hand that one run to the worker (Figure 6b). If a worker is available and all queued simulations have been performed, then the manager will task the worker with identifying how many simulations are necessary for the next configuration (Figure 6c), which will refill the job queue.

## Results

**Figure 7:**
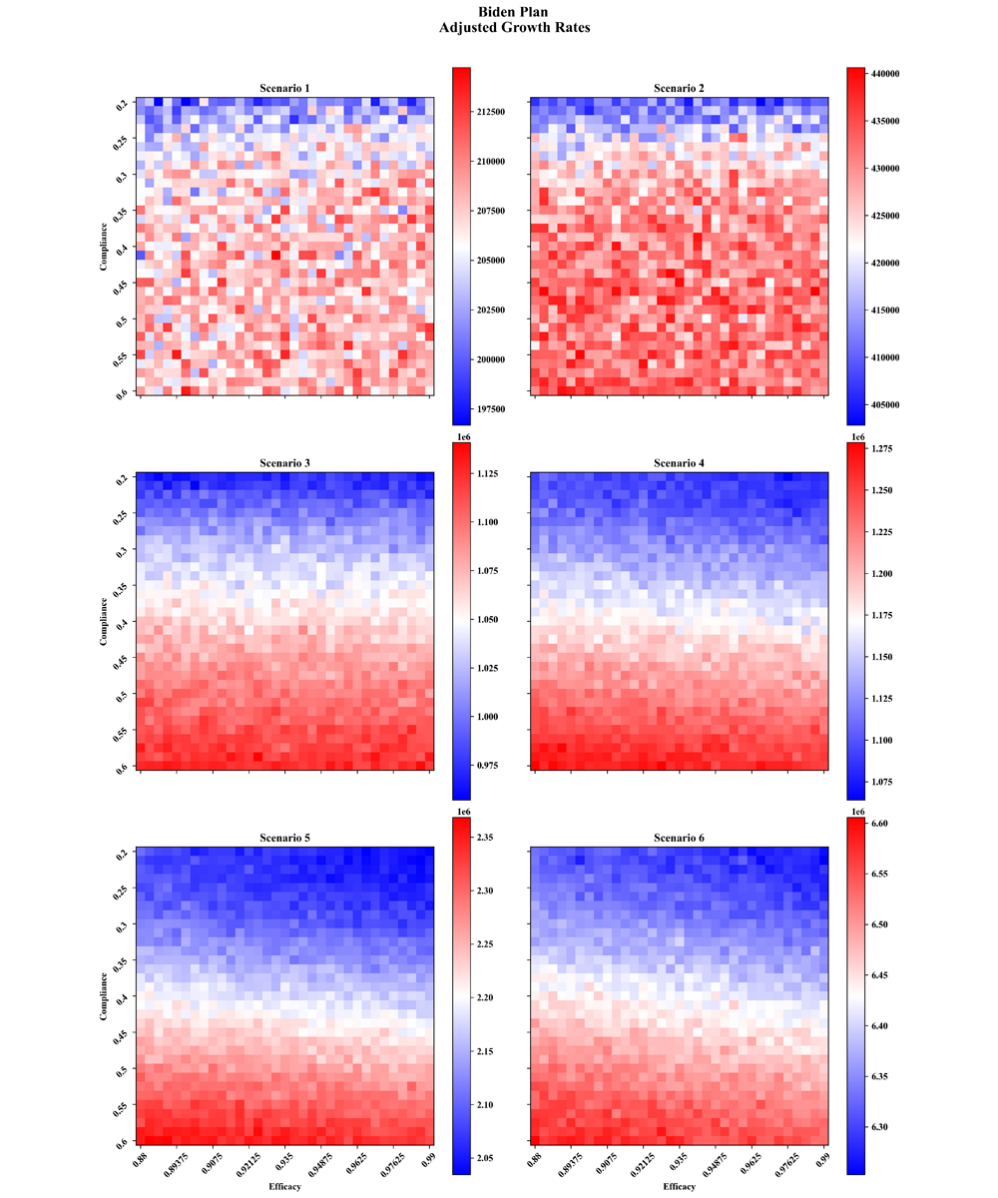
Adjusted growth rate (number of infected individuals on the worst day) as functions of vaccine compliance and efficacy under the Biden vaccination plan.

**Figure 8:**
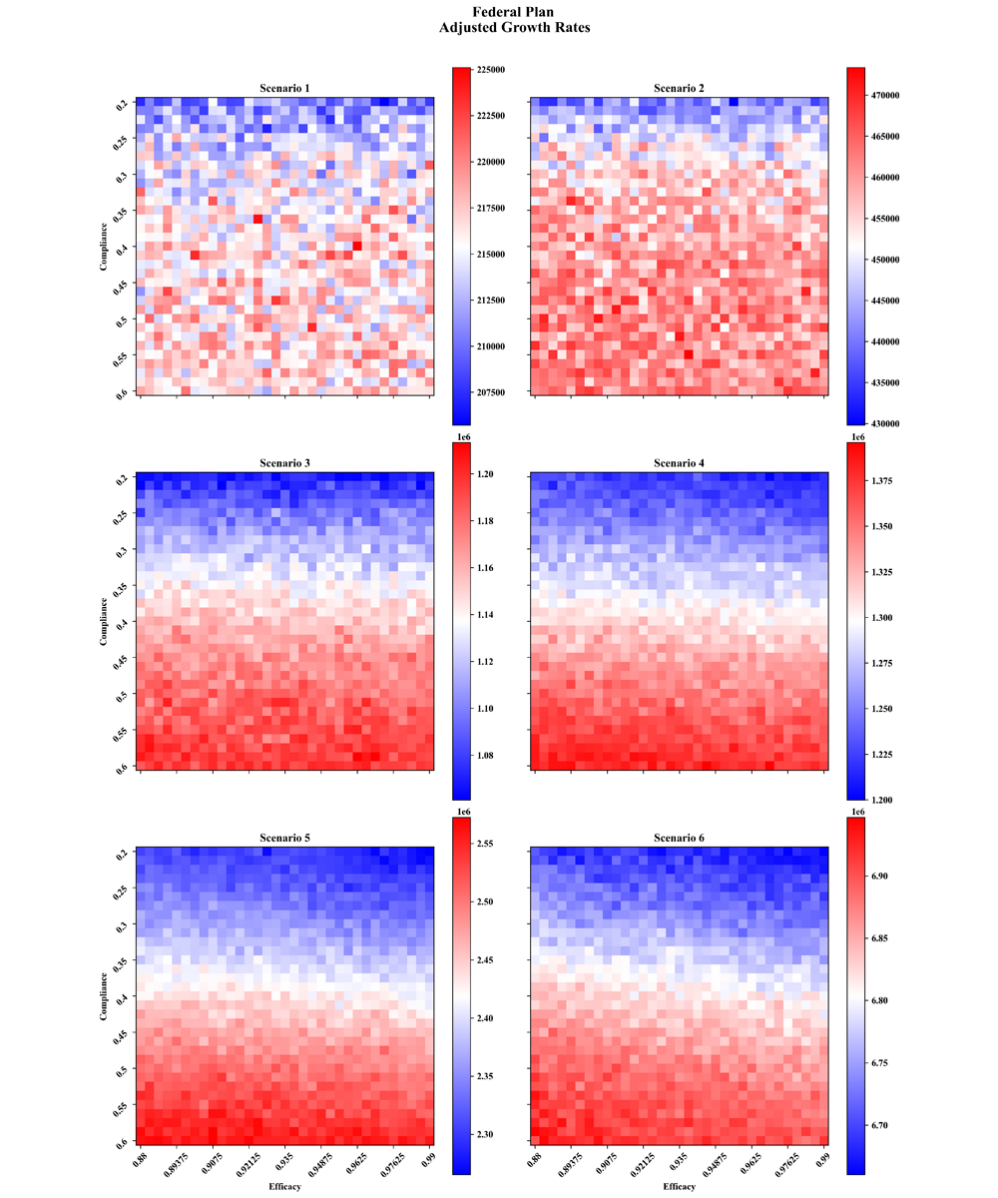
Adjusted growth rate (number of infected individuals on the worst day) as functions of vaccine compliance and efficacy under the Trump vaccination plan.

The carrying capacities and growth rates as functions of vaccine compliance and efficacies for each vaccination plan are provided in Multimedia Appendices 1-4. In this paper, we focused on the adjusted growth rate in Figures 7 and 8 for the two federal plans, 6 scenarios (including 5 nonpharmaceutical interventions), and by varying vaccine efficacy and compliance. This allowed us to examine the synergistic effects of nonpharmaceutical interventions with vaccines while comprehensively accounting for key unknowns.

In comparing the two federal plans, the Biden plan showed more potency at controlling the infection across all intervention scenarios than the plan created under the previous administration. We noted that even if a small fraction of the population seeks vaccines, and even if vaccines are less effective than announced, the vaccination campaign can reduce the total number of infections. Note that increasing the efficacies of vaccines results in lower infections for all scenarios and vaccine plans. This agrees with expectations since, in our simulations, agents are not revaccinated upon having no immune response. Therefore, holding all else equal, increasing the vaccine efficacy accelerates the growth of the immune population, thereby reaching herd immunity more quickly. In contrast, the dependence on compliance is much less intuitive and even leads to unintended consequences.

Typically, we assumed that higher vaccine compliance will lead to lower overall infections, since the proportion of the immune population is upper bounded by the compliance. However, in both vaccination plans, only scenarios 1 and 2 yielded such results. For the rest of the scenarios (3-6), the dependence on vaccine compliance is apparently reversed, with some hinting toward a nonmonotonic relationship (scenario 4 of the Biden plan and scenario 5 of the federal plan, for example). The reason behind this puzzling behavior is a combination of three factors: (1) vaccines are strictly administered in decreasing order of age; (2) older adults are going neither to work nor to school, hence they have fewer social ties than other age groups, which reduces their impact on preventing the spread of infections once immunized; and (3) relative to the growth of infections in the scenarios in which the anomaly happen, the vaccine availabilities are too low.

If we assume that an increase in vaccine compliance at the population-level is approximately uniform across age categories, then a rising vaccine compliance means that more older adults will seek vaccines. If they are also given priority for vaccines, then an increase in vaccine compliance will lead to more doses being used by older adults, hence more time to provide access to younger age groups. In short, under a vaccination strategy that focuses on older individuals, an increase in vaccine compliance will increase the delay before the more connected and younger age groups can be vaccinated. During this time, the virus can continue to spread among the younger population, particularly because the scenarios with counterintuitive results (3-6) are among the least restrictive in terms of nonpharmaceutical interventions and older adults have a lower contribution to the spread of infections due to their more limited social ties. Therefore, although the older adult population will be better protected, the longer delay for the rest of the population means that by the time they are eligible for vaccinations, the infection has already spread, leading to overall higher infections.

This argument is most vividly illustrated by our animations in Multimedia Appendices 3 and 4, in which the distributions of the infected and immune population are plotted at each time step. These animations showcase the no-intervention scenario (scenario 6) and Operation Warp Speed for the monthly vaccination capacity. Apart from the compliance, every other parameter including the random seed is fixed to be the same. Particular attention should be paid to the spread of infection among the older adult agents (ie, 65 years and older), as it most directly corroborates the aforementioned reasoning.

## Discussion

### Principal Results

The incoming CDC director predicted half a million deaths by mid-February 2021 [56], thus stressing the urgency of vaccination. However, vaccination is an unprecedented and complex endeavor whose success depends on many other variables such as vaccine compliance, vaccine efficacy, and the ongoing presence of nonpharmaceutical interventions. In line with expectations, our large-scale agent-based simulations showed that vaccination can reduce the total number of infections across all possible scenarios. The capacity pledged under the new Biden plan (one million doses a day) would have a greater impact than the plan of the previous administration (*Operation Warp Speed*) when accounting for its initial delays.

Two key findings of our study are as follows. First, we demonstrated the necessity to maintain nonpharmaceutical interventions over the next 6 months. As interventions are relaxed (from scenario 1 offering the most control to scenario 6 offering no control), there is an increase in case count such that a return to normalcy is not achieved through vaccination but rather through a very high number of infected individuals. Second, there is an unexpected interplay between vaccination strategies, nonpharmaceutical interventions, and vaccination availabilities. As nonpharmaceutical interventions lose momentum (scenarios 3 and above), an increase in vaccine compliance leads to an unexpected increase in infections due in part on the low availability of vaccines and the priority on vaccinating older adults. More so than the observation that tighter nonpharmaceutical interventions result in the slower spread of infections, this result further delineates the necessity of preparing the population to continuing nonpharmaceutical interventions even as the vaccination progresses.

### Limitations

There are three main limitations to our current understanding of the COVID-19 pandemic and the vaccination campaign that affect how our simulations account for (1) the number of vaccines that *can* be administered each month, (2) biological aspects, and (3) *healthy* or asymptomatic carriers.

First, an unprecedented vaccine campaign comes with logistical challenges and uncertainty given the complex array of factors involved. As a result, fewer than the expected number of doses may be administered: federal officials aimed at giving the first dose to 20 million people during December 2020, but various delays resulted in fewer than 3 million people receiving a first dose [57]. It was recently reported that “federal officials say they do not fully understand the cause of the delays” [57] and that the administration “pledged to immediately distribute millions of COVID-19 vaccine doses from a stockpile that the U.S. health secretary has since acknowledged does not exist” [58]. This situation has resulted in views that “much of the narrative earlier this year regarding Warp Speed’s preparation appears to be a sham” [59], reinforced by reports that the Biden administration found no vaccine distribution plan upon taking over from their predecessors [60]. Some of the factors causing a delay are known: there can be shipping delays or delays in administering doses due to a lack of hospital staff members, as they are already caring for individuals infected with COVID-19. Other factors may be more surprising, such as the intentional destruction of vaccine doses by hospital staff [61]. As any simulation model is necessarily a simplification, we did not include factors whose value would be entirely unknown (eg, what will be the shipment delay?) or whose existence is anecdotal given the total number of doses (eg, intentional destruction or storage errors). We were limited in our ability to use real-world numbers on how many individuals received the vaccine, as this data is captured at the state level, and several states’ reporting systems have experienced errors [62]. Although there are efforts at centralizing data (eg, national news outlets aggregate data across states [63]), the level and nature of errors differ across states, which is a challenge to estimate overall model uncertainty.

We have thus followed the federal plan for the number of individuals who can get vaccinated each month. Out of all the doses that are *planned*, fewer may be *distributed* and an even lower number may ultimately be *administered*. Our simulations are thus likely representing an upper bound on the number of vaccines administered, leading to *more optimistic results than in reality*. The gap is particularly pronounced in December 2020 and may remain significant in January 2021, but early logistical issues and delays should be gradually addressed, such that the gap between federal expectations and actual implementation narrows over time.

Second, all biological aspects of the virus are based on the strains that dominated throughout 2020. Epidemiological studies from these strains have informed parameters such as transmissibility, incubation period, the proportion of asymptomatic carriers, the severity of symptoms and hence the course of the disease, and the efficacy of treatments or vaccines. The existence of different strains is well established, as phylogenies have shown seven distinct lineages [64,65], but there has not yet been a documented need to ascribe different parameter values (ie, different viral *behaviors*) to each strain. There are two possible reasons. First, there are relatively few mutations and thus a limited *chance* of a drastically different outcome naturally occurring: the virus is “considered a slowly-evolving virus as it possesses an inherent proofreading mechanism to repair the mismatches during its replication” [65]. Second, there has been little selective pressure on the virus, as it was spreading through a population that had never been exposed to an antigen (ie, immunologically naïve). Both arguments are now changing.

A new strain from the lineage B.1.1.7, named Variant of Concern 202012/01 (denoted VOC-202012/01), emerged with an unusually large number of 23 changes in its genomes (including mutations and deletions) [66]. Some of the biological changes make it easier for the virus to attach to its targets and enter cells, which is captured through epidemiological indicators as increased transmissibility [67,68]. This is relevant for our study, as this more contagious COVID-19 strain has been spreading in the United States and may dominate by March 2020 [69]. To date, there is no peer-reviewed evidence of an impact on disease severity or vaccine efficacy over a large population sample, but the function for some of the mutated parts remains unknown (hence the possibility of an impact on severity), and early studies over 20 volunteers suggest that antibodies from vaccines are only one-third as effective on some variants [70]. In addition, vaccination means that the virus is no longer spreading through an immunologically naïve population, thus creating selective pressure for functional mutations that can help the virus adapt. Our *simulation results are thus optimistic* as they use a lower transmissibility than provided by the new strain, and we did not worsen any of the other parameters to account for possible selective pressure.

Third, our model considers that individuals who were successfully immunized can act as a buffer in the spread of the epidemic. Reality may be more nuanced, as viral transmission from a vaccinated host to an unvaccinated one may be possible. At the time of writing (March 2021), we do not yet have conclusive findings about this possibility. As trials continue, we may find that immunized individuals should be treated in a model as *healthy carriers* for a period. We also noted that the immunity conferred by the vaccine appears to have a different response than the immunity acquired by recovering from a natural infection. That is, a vaccine promotes the production of antibodies in the blood, but a natural immunity may lead to developing antibodies in the mucosal regions [71], which are the first site of infection (in the nose and mouth). From a modeling viewpoint, the two immunities may thus have to be treated differently in the future.

Finally, we note that our model is *built specifically for the United States*. It would not be accurate when transposed to another country with minimal changes (eg, only reducing the population size). For example, stark differences in vaccine rollout strategies exist between the United Kingdom and the United States, which would affect our simulations. In the United States, two doses of the same vaccine are normally administered, as the CDC stated that “mRNA COVID-19 vaccines are *not* interchangeable with each other or with other COVID-19 vaccine products” [72]. However, new guidance from the United Kingdom allows a mix-and-match vaccine regimen in which the second dose may be from a *different* vaccine in exceptional circumstances (eg, if the vaccine from the first dose is not available upon the patient’s return), even though clinical trials for mixed regimens are only due to be conducted at a later, unspecified time [73]. Another difference is that the United Kingdom front-loads the vaccine by delivering *as many first doses* as possible, which thus no longer guarantees that a patient can receive the corresponding second dose upon return (hence raising the need for a mix-and-match) and potentially delays the delay before a second dose up to 12 weeks [73]. In contrast, the United States is against delaying the second dose [74], thus our model operates on the assumption that a patient can complete treatment on time.

### Related Works: The Scale of Agent-Based Models for COVID-19

Our simulation of half a million agents qualifies as large-scale *in the context* of COVID-19 ABMs. In another context, the scale may be different as the computational costs of the simulation or historical practices in a research community may differ. For example, in HIV research, simulations have used half a million cells for about 20 years on personal computers, so a *large-scale* may be a more appropriate qualifier for simulations with billion cells [75,76]. As noted by Gumel and colleagues [77] in their extensive discussion on modeling methods for COVID-19, ABMs “are computationally-intensive”; thus, we may expect a smaller simulated population than in compartmental models or meta-population models, given the same hardware and simulation time.

Many ABMs for COVID-19 are in the scale of several hundred agents [78-83] to tens of thousands of agents [37,84,85]. Fewer studies have over 100,000 agents [86], and only a paucity of studies has a number of agents that is about equal (eg, the model of Hoertel and colleagues [87] used 500,000 agents) or greater than (eg, one million agents in a February 2021 simulation of Bogota) in this study [38,87,88]. Due to this distribution of agent population across studies, the qualifier of *large* is applied as we get to the scale of 500,000 or more agents [38]. It should not be interpreted to suggest that this is the *largest* population size achieved to date. Indeed, a few high-profile studies have modeled their target populations with such a fine resolution that the simulation may qualify as a *digital twin*. For example, Chang et al [89] used over 24 million agents by adding a COVID-19 component (AMTraC-19) to an existing model and running it over 4264 compute cores.

Although a justification for the scale is a recommended best practice in ABM for artificial societies [90], such a justification is not always present in published studies. The studies that have justified their choice of scale have often done it based on the size of the target population (eg, single city or campus) or implicitly invoked the notion of a computational burden when downscaling. Explicit mentions of computational costs have been made by the developers of frameworks, such as Comokit, who stated that 10-20,000 agents could be simulated on one laptop within 10 minutes [37].

### Conclusions

A desirable return to normalcy would be achieved via immunization rather than through a very high number of infected cases and their natural immunity. Our extended ABM shows that vaccines are not sufficient to return to normalcy while avoiding a high number of cases. Nonpharmaceutical interventions are necessary and require a high level of compliance to ensure that immunity from vaccination outpaces the immunity from infections. Although our findings account for different vaccination capabilities, compliance levels, and vaccine efficacy, they are nonetheless based on a simulation model, which is necessarily a simplification of reality. Simplifications here include the logistics of vaccine dissemination, variants, and the presence of *healthy carriers* (vaccinated) and asymptomatic cases (not vaccinated).

## Appendix

Multimedia Appendix 1 Biden carrying capacity.

Multimedia Appendix 2 Operation Warp Speed carrying capacity.

Multimedia Appendix 3 High compliance.

Multimedia Appendix 4 Low compliance.

## Abbreviations

ABM: agent-based model
CDC: Centers for Disease Control and Prevention
