# Supplementary figures and images for: Returning to a Normal Life via COVID-19 Vaccines in the United States: A Large-scale Agent-Based Simulation Study
Source: JMIR Med Inform. 2021 Apr 29;9(4):e27419. doi: 10.2196/27419 (PMC8086790; doi:10.2196/27419)

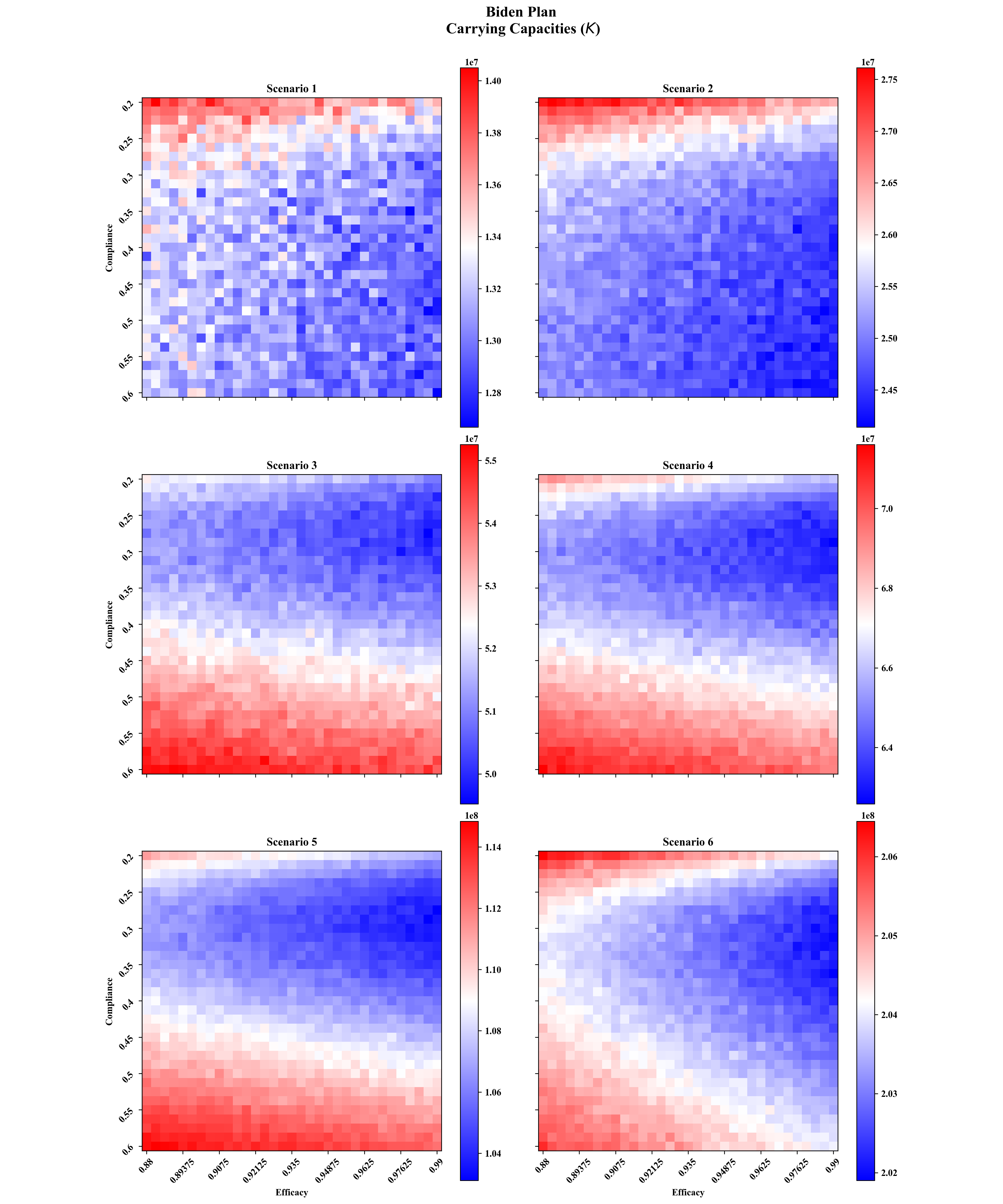

Supplement: Multimedia Appendix 1 [file medinform_v9i4e27419_app1.png]

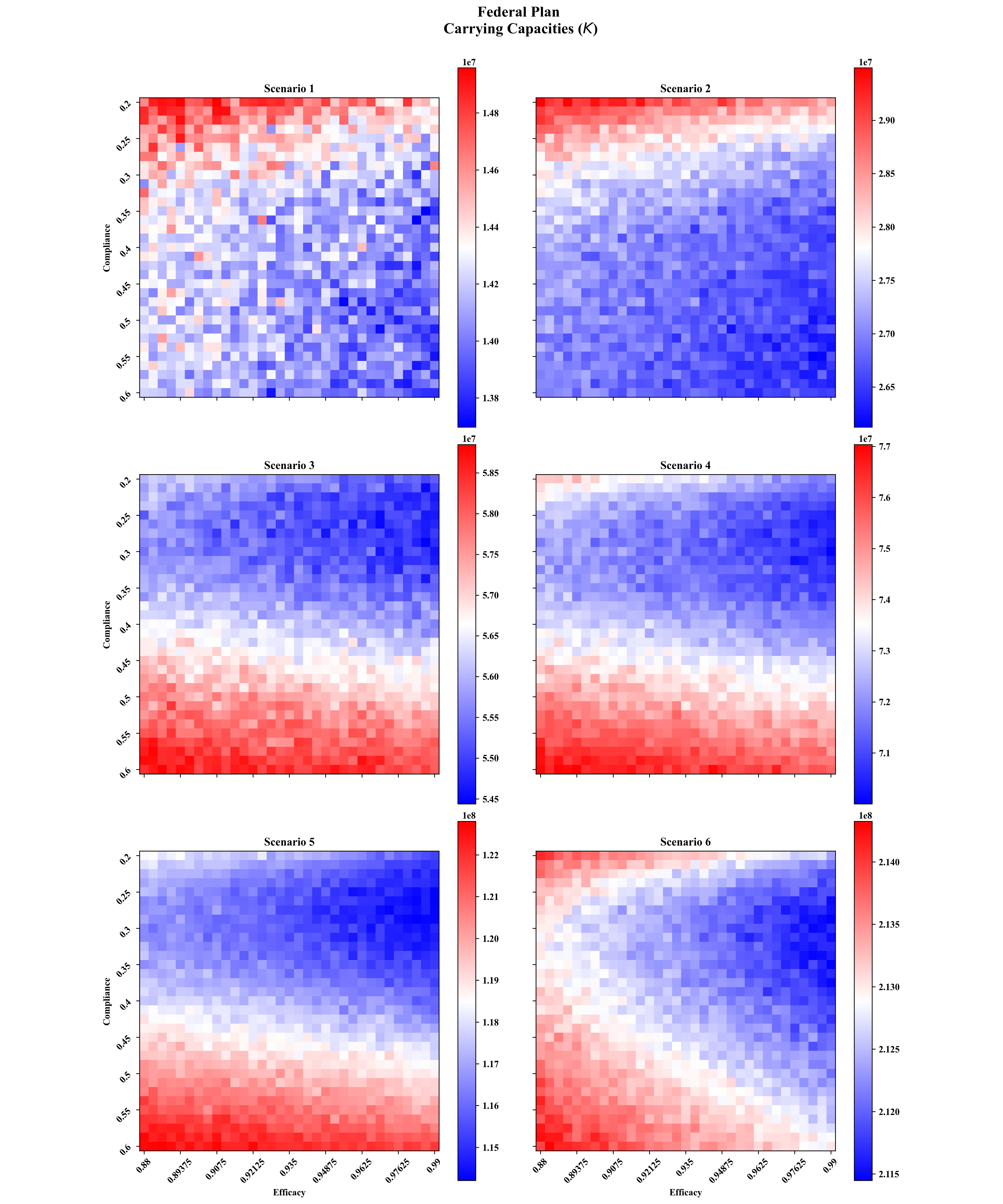

Supplement: Multimedia Appendix 2 [file medinform_v9i4e27419_app2.png]
